# Discrimination, Microaggressions, and Perceptions of Institutional Response in an Academic Obstetrics and Gynecology Department

**DOI:** 10.7759/cureus.15993

**Published:** 2021-06-28

**Authors:** Huma Farid, Hannah Stack-Dunniber, Rose Molina, Catherine Nosal, Monica Mendiola, Michele Hacker

**Affiliations:** 1 Obstetrics and Gynecology, Beth Israel Deaconess Medical Center, Harvard Medical School, Boston, USA; 2 Obstetrics and Gynecology, University of Massachussetts, Worcester, USA; 3 Obstetrics and Gynecology, Northwestern University Feinberg School of Medicine, Chicago, USA

**Keywords:** discrimination in health care, microaggressions, gender bias, racial bias, implicit bias

## Abstract

Introduction

Discrimination in the workplace remains a barrier to advancing diversity and inclusion in the physician workforce. This study sought to examine experiences of discrimination, microaggressions, and perceptions of the institution’s response in an academic obstetrics and gynecology department.

Method

All obstetrics and gynecology faculty, fellows, and residents were invited to complete an anonymous, Institutional Review Board-approved cross-sectional survey from February through June 2019. The survey incorporated questions from multiple validated tools on discrimination, microaggressions, perceptions of the institution’s response, and opportunities for comments. Data are presented as the frequency with percent and were analyzed using Stata (StataCorp, College Station, USA); two of the authors reviewed and deductively coded the qualitative data.

Results

The response rate was 58% (87/151), with 30% of the respondents identifying as trainees and 75% identifying as female. Thirty respondents (35%) identified as non-Caucasian. Fifty-four respondents (62%) had ever experienced discrimination and 63 (72%) reported ever experiencing microaggressions at work; of those, 14 (22%) experienced microaggressions several times per week. Of the 69 respondents (79%) who experienced microaggressions and/or discrimination, 49 (71%) felt their experiences were due to gender, and 26 (38%) felt that they were due to race/ancestry. Only 41 respondents (59%) felt that the institution was fair to all employees, and 17 (25%) did not believe diversity was managed effectively.

Conclusion

Most physicians in the department experienced microaggressions or discrimination, with gender or race/ancestry as common inciting factors. A small but notable portion of respondents would prefer the institution to manage diversity differently. These findings merit further investigation about how to address discrimination in academic medicine.

## Introduction

Multiple national studies have demonstrated significant rates of discrimination and harassment in medicine, ranging from 32% [1] to 93% [2] of physicians ever having experienced discrimination or harassment. Female physicians and non-White physicians are the most frequent targets of discrimination [1]. A national study of trainees in surgical residency demonstrated that 65% of female surgical residents had experienced gender-based discrimination [1]. Physicians who are people of color (used as an inclusive term referring to all racial groups that are non-white [3]) encounter discrimination based on race/ethnicity. The majority of physicians of color experienced discrimination at work, [4] with Black physicians reporting the highest rates of discrimination (71%) [4,5]. These experiences occurred at all levels of their education, often starting before medical school [6].

While overt discrimination in medicine is rare, microaggressions are common [7]. In this study, we defined microaggressions as “a comment or action that subtly and often unconsciously or unintentionally expresses a prejudiced attitude toward a member of a marginalized group” [8] and discrimination as “a prejudiced outlook, action, or treatment” [9]. There is growing awareness that unconscious bias permeates the workplace and may lead to a culture of microaggressions [10]. These experiences impact physician retention and job satisfaction: 25% of physicians of color attributed switching jobs due to workplace discrimination [4]. Physicians who experienced discrimination felt that it impacted career advancement, felt less welcome at work [11], had difficulty finding mentorship, believed that their race influenced work relationships [5], and had higher rates of depression and suicide attempts [12,1].

The specialty of obstetrics and gynecology is unique in that it has a larger proportion of women than other areas of medicine; 81% of obstetrics and gynecology residents are women, which is the largest proportion of female residents in any residency program in the country [13]. However, Hofler et al. demonstrated that despite boasting a larger proportion of women in the field, only 20% of obstetrics and gynecology department chairs were women, and women were underrepresented in all leadership roles within departments with the exception of residency program directors and medical student clerkship directors [14]. When compared to other specialties, although obstetrics and gynecology departments had the largest proportion of female leaders, the representation ratios lagged behind other specialties given the large number of women in the field [15].

Our study examined the rates of discrimination and microaggressions in an obstetrics and gynecology department and evaluated perceptions of the institutional response. Given the breadth of studies demonstrating significant rates of discrimination and microaggressions among physicians, our study intended to assess whether gender and race played a role in a female-led and female-majority obstetrics and gynecology department. This study also examined perceptions of the institution among physicians who had experienced discrimination and microaggressions.

## Materials and methods

This cross-sectional, online, anonymous survey on discrimination and microaggressions in the workplace was sent to all physician members of the Department of Obstetrics and Gynecology at a tertiary care hospital in a major urban center in the Northeast. The study was deemed exempt from the Institutional Review Board and received approval. We sent invitations to complete the anonymous survey via email from February through June 2019. We adapted survey items from validated survey tools described below, and participants could also write in additional comments within the survey. The survey is included in the supplemental content. We administered a pilot survey to four members of the department and revised it based on their feedback.

The survey included basic demographic questions, asked physicians whether they identified as a minority in the field and why, and whether they were familiar with the provided definition of microaggression. We specifically inquired whether respondents “identified as a minority in the field” in order to capture those respondents who are not traditionally thought of as underrepresented in medicine. For example, although men are not underrepresented in medicine, currently there are more women entering the field of obstetrics and gynecology than men [13], effectively making men a minority in the field. Respondents were then asked if they had ever experienced microaggressions or discrimination, the frequency of these experiences, the identity of the transgressors, and why they believed they experienced discrimination or microaggressions (the choices offered included: ancestry, physical appearance, gender, sexual orientation, race, educational level, physical disability, age, or religion). Respondents were asked how often they had experienced a patient refusing to be examined by them and how often they were told they appeared too young to be a physician.

The survey also included 15 questions adapted from the Diversity Engagement Survey from the American Association of Medical Colleges. This survey examined eight engagement and inclusion domains. Each domain mapped out to several questions that form the survey’s framework [16]. We adapted questions from six out of the eight domains that we thought most relevant to our institution and that focused specifically on the institution’s environment of engagement, inclusion, and diversity: cultural competence, common purpose, equitable reward and recognition, sense of belonging, trust, and respect. These 15 questions evaluated the institution’s ability to demonstrate cultural competence and inquired about a shared common purpose among employees at the institution, equity among the financial arenas of the institution, employees’ sense of belonging and trust within the institution, and institutional respect for diversity. Specifically, physicians were asked whether they felt that the institution was fair to all employees, whether they felt connected to the mission of the institution, whether they had opportunities to work with diverse colleagues, and whether the institution would equitably address concerns regarding discrimination.

A series of eight questions were asked about respondents’ treatment from coworkers; we incorporated these questions from the Everyday Discrimination Scale [17]. We asked respondents how frequently they had experienced certain situations at work, such as being treated with less courtesy and respect than others, being called names, or being threatened or harassed, which originated from the Chronic Work Discrimination and Harassment (YES) Study [18,19]. Finally, there was an open-ended option offering respondents the opportunity to include any additional information about their experiences.

Data are presented as frequency with percent and were analyzed with Stata version 14 (StataCorp, College Station, USA). Two investigators (HF, HSD) independently reviewed open-ended survey responses. They read the responses in their entirety and deductively coded them based on common themes. The investigators created a codebook with five broad categories and conducted thematic content analysis. Categories included: gender, race/ethnicity, age, socioeconomic status, and sexuality, with each of these further subdivided into subcodes. After analysis of each of the four open-ended questions, the two investigators assigned each response a subcode within a broader category. The institution’s Committee on Clinical Investigations approved this study.

## Results

Of 157 physicians, 87 (56%) completed the survey. Table 1 presents the respondents’ demographic information. Fifty-seven (66%) respondents identified as white/Caucasian, and 30 (34%) identified as non-Caucasian, including 10 (11%) as Asian, 8 (9%) as Black/African American, and 5 (6%) as Hispanic/Latinx. Sixty-five (75%) of the respondents were female. Twenty-six (30%) of the respondents were residents or fellows; 59 (68%) were faculty. The median age was 41 (IQR 33-56) years. Twenty-seven respondents (31%) self-identified as a minority in the specialty, most commonly due to race. Most physicians 67 (77%) had experienced microaggressions at work, and 59 (68%) had experienced discrimination at work. The majority of respondents (71%) who had ever experienced microaggressions or discrimination attributed these experiences primarily to their gender.

**Table 1:** Demographic Characteristics of Respondents IQR: inter-quartile range

Characteristic	Respondents N=87, n (%)
Race/Ethnicity	
White/Caucasian	57 (65)
Asian	10 (11)
Black/African American	8 (9)
Hispanic, Latinx, or Spanish	5 (6)
Multiracial	5 (5)
Other	3 (3)
Gender	
Female	65 (75)
Male	22 (25)
Level of training	
Resident	21 (24)
Fellow	5 (6)
Attending	59 (68)
Missing	2 (2)
Median Age	41 (IQR, 33-56)
Identify as minority in specialty	
Yes	27 (31)
No	59 (68)
Reason for self-identification as a minority	
Race	17 (20)
Ancestry or national origin	14 (16)
Gender	9 (10)
Appearance other than height/weight	6 (7)
Religion	5 (6)
Sexual orientation	2 (2)

**Figure 1:**
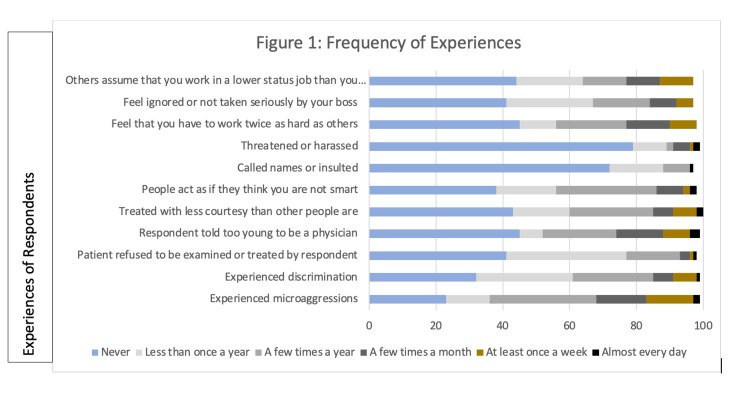
Frequency of Experiences

In terms of frequency, 14 physicians (16%) experienced microaggressions at least weekly to nearly daily, and seven respondents (8%) experienced discrimination at least weekly to nearly daily. Fifty-one physicians (59%) had ever had a patient refuse to be examined or treated by them; 17 (20%) reported this occurred a few times a year or a few times a month, and two (2%) reported this to be a weekly or daily occurrence. Forty-one respondents (47%) had been told that they appeared too young to be a physician. One physician had been asked by multiple patients if they were “experienced enough to care for them.” The majority of physicians (56%) attributed these particular experiences of microaggressions to gender, followed by ancestry/national origin or race (30%). One respondent described “patients have asked to be examined by more junior residents or medical students who are male.” Both men and women faced gender bias; a physician reported that “patients will refuse a male provider for religious or personal preference.” Figure 1 illustrates the respondents’ experiences with microaggressions and discrimination.

Physicians identified patients as the most frequent transgressors (61%), although nearly every person a physician could encounter in the hospital (including medical students, nurses, support staff, nurse practitioners, surgical technicians, and other physicians) was also responsible. More than half of respondents (59%) had not informed anyone of their experiences, but 29 (33%) indicated that they confided in a colleague.

The survey also examined the environment of the workplace. Twenty-two physicians (25%) reported being treated with less courtesy than others a few times a year; five (6%) reported experiencing this a few times a month, and six (7%) felt that they were treated with less courtesy than others at least weekly. Fifty-four physicians (62%) had felt at least once that people acted as though they were not smart. One physician reported that “individuals [were] voicing surprise that someone like me can be a gynecologic surgeon, that I am so articulate.” Twenty-four respondents (28%) had been called names or insulted, and 18 (21%) had been threatened or harassed. Thirty-six respondents (41%) felt that they had to work twice as hard as others, and 26 (30%) felt that they were not taken seriously by their supervisor. A respondent stated a “supervising physician asked me why I work so hard as I’m married and my husband can take care of me.” Another physician shared, “As a female moving into leadership roles, I am often asked why I am present at meetings.” Twenty-nine respondents (33%) experienced others assuming that they were not physicians. Of those physicians who had experienced these behaviors, 47% attributed these experiences to gender, followed by age (26%) and ancestry/national origin or race (25%).

The analysis of qualitative data revealed five major themes that recurred in physicians’ free-text responses providing examples of discrimination: gender, race/ethnicity, socioeconomic status, age, and sexuality. Respondents described gender-based discrimination most frequently, with 81% of responses illustrating bias due to gender, followed by race/ethnicity (31%). Common subthemes among physicians who faced gender-based discrimination centered around pregnancy/childcare (someone asking them when they would stop pumping), unequal opportunities in the workplace (being questioned about their presence at meetings and salary disparities), and being a male physician in obstetrics and gynecology (patients declining to have a male provider involved in their care). Microaggressions such as women physicians being misidentified as non-physicians or junior physicians commonly occurred.

Physicians who encountered discrimination based on their race/ethnicity described frank bias (being told that they have “small Asian fingers”), overt disrespect (“name calling” and the use of “disrespectful language”), and attitudes of indifference to their input as subthemes. Microaggressions, such as being told they were “so articulate,” questioning their surgical ability, and “being called by the wrong name,” were pervasive. Physicians who experienced age-based discrimination described that patients questioned their experience because they looked young. One physician reported “being told [that I am] too young to be in leadership.” Reports of discrimination due to socioeconomic status and sexuality were less common but were no less powerful. One physician described that when the default culture emphasized heterosexuality, others assumed they were “married to a person of the opposite sex.” Table 2 summarizes common themes and representative comments.

**Table 2:** Examples of Discrimination in the Workplace, n=26 OBGYN: obstetrics and gynecology

Theme and subthemes	Frequency (percent)*	Representative Quotes
Gender	21 (81)	“Supervising physician asked me why I work so hard as I'm married and my husband can take care of me.” “After introducing myself as "Dr. __" and reviewing and counseling about results with patient and husband, they asked when the (male) doctor would be seeing them.”
Pregnancy/childcare	2 (10)	“Was asked by an attending when I was ‘going to stop the whole pumping thing’ when I said I couldn't make lunch with everyone between back to back OR cases.”
Unequal opportunities in the workplace	4 (20)	“As a female moving in to leadership roles I am often asked why I am present at meetings.” “My pay is lower than all men in my position with my years of experience.” “I was told I was too young to be in a leadership role.”
Microaggressions	3 (14)	“Male staff or patients or family members assume I'm not a doctor because I'm female.” “Staff has addressed more junior staff who are male directly with questions or suggestions and bypassed me.” “Pretty sure if a male MD were asking there would be less eye-roll and passive aggressive pushback.”
Male in OBGYN	7 (33)	“Patient elected not to have a male provider. Patient often say to me, I thought I was going to see a female doctor.”
Race/ethnicity	8 (31)	“I've had nurses ask me to translate for them because they did not want to call the interpreter.” “Minorities are questioned and challenged more than the non minorities in the department by support staff.”
Racial/ethnic bias	4 (50)	“A nurse has also told a patient in front of me that I have small Asian fingers so my vaginal exams are more comfortable than from other providers.”
Overt disrespect	1 (13)	“Disrespectful language/name calling.” “I've been told that I have no eyelids.”
Microaggressions	3 (38)	“Over penalization for minor offenses.” “Ignoring accomplishments/accolades.” “Individuals voicing surprise that *someone like me can be a Gyn surgeon. *that I am so articulate. Being called by the wrong name.”
Indifference	1 (13)	“Dismissive of colleague input.”
Age	6 (23)	“Patient thinking I am too young to be a physician and asking for an attending for exam.” “Being told too young to be in leadership.” “Frequent comments about too young to be a surgeon from patients.”
Assumption of inexperience	3 (50)	“Multiple patients have asked me if I am experienced enough to care for them, if I'm really the physician.”
Microaggressions	3 (50)	“Patients will ask how many of a particular procedure I have done.”
Socioeconomic status	1 (4)	“On L&D, RNs and MDs provide differential care to affluent, well educated white patients as compared to resident clinic patients and those from the community health centers, which tend to be poorer, less educated, with fewer resources, and less white.”
Sexuality	1 (4)	“Assumptions of being married to a person of the opposite sex.”

We examined the experience of microaggressions and discrimination comparing white and non-white female respondents. Non-white female respondents were more likely to experience microaggressions than white female respondents (p=0.012); these differences were not statistically significant for experiences of discrimination or microaggressions and discrimination combined (p=0.074). When we analyzed responses from all 69 respondents who had experienced either microaggressions or discrimination, 49 (71%) felt their experiences were due to gender, and 26 (38%) felt that they were due to race/ancestry.

**Figure 2:**
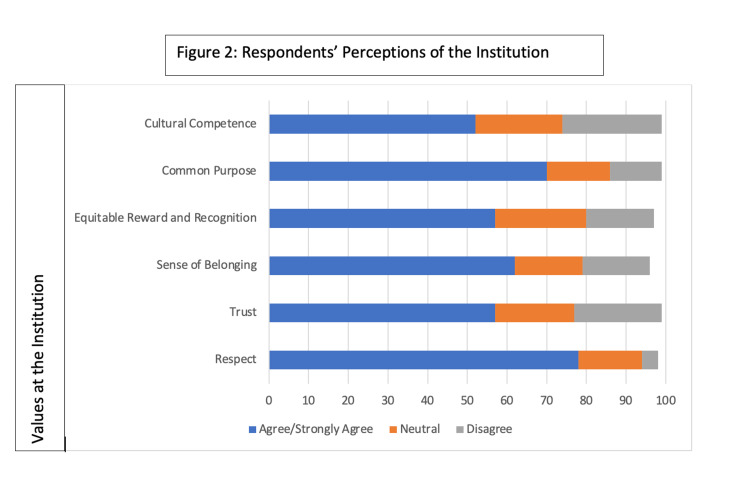
Respondents' Perceptions of the Institution

We subsequently analyzed the responses to the questions adapted from the Diversity Engagement Survey to assess attitudes toward and perception of the institution from physicians who had experienced microaggressions or discrimination. Of the 69 physicians who had experienced microaggressions or discrimination, the majority (78%) agreed that they encountered respect when working with diverse colleagues and 48 respondents (70%) felt connected to the vision and mission of the institution (thereby aligning with the domain of a shared common purpose) despite their experiences. However, questions that mapped to the domain of trust demonstrated different results. Out of the physicians who had experienced microaggressions or discrimination, only 41 respondents (59%) felt that the institution was fair to all employees, and even fewer respondents - 39 physicians (57%) - felt confident in the institution’s ability to handle discrimination. Similarly, only 57% of respondents felt that they received equitable rewards when compared to others, and only 43 physicians (62%) felt a sense of belonging within the institution. Questions that mapped to the domain of cultural competence were notable for the fact that while the majority of respondents (70%) indicated that they had opportunities to work with diverse colleagues, fewer felt that the institution’s management or support of cross-cultural groups was sufficient. For example, seventeen respondents (25%) did not believe diversity was managed effectively, and only 41 (59%) felt supported in working in a cross-cultural context. Figure 2 illustrates respondents’ attitudes toward and perception of the institution.

## Discussion

In a relatively diverse obstetrics and gynecology department, the majority of physicians have experienced discrimination or microaggressions at least once in the workplace, which is similar to other published studies [1-5]. Physicians mainly attributed these experiences to gender, followed by race, which could be due to the fact that we had more respondents who were female than people of color. In a recent survey of surgery residents, more respondents experienced gender-based discrimination than race-based discrimination (31.9% and 16.6%, respectively) [1], which correlated with our findings that more respondents experienced gender-based discrimination. These findings of gender-based discrimination are notable in a majority female department (75% of physicians were female) and the majority of whose patients identify as female. In addition, there was some evidence that race compounded the effects of gender-based microaggressions for respondents who were not white. The intersection of identities is inevitable as the workforce becomes more diverse, and discrimination based on gender and race may be compounded in these situations.

Another relevant finding was that physicians experienced discrimination and microaggressions from nearly every person they encountered at the workplace, including patients, colleagues, nurses, medical students, support staff, and scrub technicians. This finding is concerning because it demonstrates that discrimination is ubiquitous and has the potential to saturate each human encounter. Furthermore, 59% of physicians did not inform anyone about their experiences, leaving little opportunity for institutional changes to be implemented.

Among physicians who had experienced discrimination or microaggressions, a small but notable portion had striking responses that demonstrated a lack of trust in the institution. These physicians felt that the institution was not fair to all employees, that harassment was tolerated, and that the institution should manage diversity differently. These physicians also did not feel that there was equity in recognition of their work when compared to others and that while the institution offered them opportunities to work with diverse colleagues, it provided very little institutional support to make those interactions successful. While an exact correlation between experiences of discrimination and mistrust of the institution is difficult to argue, physicians’ personal experiences may have negatively affected their perceptions of the institution.

In addition to reporting discrimination and microaggressions, this study has a novel approach in that it also examines institutional perceptions among a cohort of physicians who had experienced discrimination or microaggressions. Employee engagement in an institution is more likely when employees’ values align with institutional values and mission [20]. In an environment where the majority of physicians experienced discrimination or microaggressions in a variety of settings in the workplace, it is then understandable that those physicians expressed a sense of mistrust in the institution and questioned the institution’s ability to support cultural competence in a meaningful manner. Similarly, a study examining an internal medicine department at an academic center found that female physicians reported higher rates of discrimination and harassment than male physicians and that they were also more likely to report less gender equity in the institution than their male counterparts [21]. Likewise, in our study, the respondents’ experiences of discrimination and microaggressions may have influenced their perceptions of the institution’s fairness and its ability to address discrimination.

The limitations of our study include a small sample size of a single department; this may limit generalizability to other departments and institutions. However, our results are similar to what larger studies have found in terms of incidence of discrimination. Particularly given the large, multi-site study recently published by Hu et al. [1], our results suggest that these findings are not related to an institution but rather are representative of the larger issues within medicine and accurately reflect physicians’ experiences across the nation. Another limitation is that while we used validated survey tools, we adapted them to fit our needs, and there could potentially be variations in how the respondents understood the survey questions. In addition, the generalizability of our results may be limited by the 56% response rate. It may be that respondents were more likely to have experienced microaggressions and discrimination. Conversely, there is also a possibility of under-reporting. We did not examine physician turnover or job satisfaction, although previous studies have demonstrated that experiences of discrimination lead physicians to leave their jobs and even leave medicine [4]. Finally, we were not able to directly determine causality or correlation between experiences of discrimination or microaggression and perceptions of the institution. 

## Conclusions

Discrimination and microaggressions are common experiences for physicians, and this study revealed that gender and race-based discrimination are prevalent in an urban, academic obstetrics and gynecology department. Physicians experience discrimination and microaggressions from a breadth of people with whom they interact daily: administrators, supervisors, colleagues, nurses, patients, and support staff. The results of our study demonstrated that experiences of microaggressions and discrimination have a significant effect on physicians’ perceptions of the institution, particularly when they felt as though the institution did not respond in a satisfactory manner to their experiences of bias. Of all the questions about institutional perceptions, the ones asking respondents about the institution’s ability to handle diversity and their trust in the institution to handle reports of discrimination effectively were the ones with the least favorable responses. When faced with this data, it is clear that simply hiring more diverse staff does not alleviate issues of equity, and that diversity alone does not equate to inclusion. Rather, the institutional leadership must actively build a culture of inclusion through the promotion of trust, a shared sense of belonging, equitable recognition, and meaningful incorporation of diversity.

The actions of an institution’s leaders when responding to microaggressions or discrimination can have a significant positive impact on building an environment of inclusion. Institutional leadership should focus on a cogent response to concerns about bias, starting with the development of an anonymous reporting system for bias-related incidents and mandating microaggressions and implicit bias training. Institutions should consider a council focused on diversity, equity, and inclusion that is appropriately funded and whose leadership has the potential and the power to build an environment of inclusion through focus groups, community outreach, and advocacy. Few physicians reported these instances in our institution. It is imperative to create a culture of zero tolerance, provide anonymous and safe mechanisms for reporting, and foster conversations about how to be an ally in this arena. This study provides valuable insight into physician experiences and serves as an impetus to continue to promote change within the culture of medicine.
